# Jefferson College

**Published:** 1844-09

**Authors:** 


*Jefferson College.*—We are gratified to learn from the annual announcement, that this institution is in a most flourishing condition, and deserves the patronage of all who are interested in the cultivation of medical science. The city of Philadelphia affords every facility for the study of medicine, abounding in hospitals and other charities.

